# Expression of guanylate cyclase-C, guanylin, and uroguanylin is downregulated proportionally to the ulcerative colitis disease activity index

**DOI:** 10.1038/srep25034

**Published:** 2016-04-29

**Authors:** Danfeng Lan, Junkun Niu, Jiarong Miao, Xiangqian Dong, Hong Wang, Gang Yang, Kunhua Wang, Yinglei Miao

**Affiliations:** 1 Department of Gastroenterology, The First Affiliated Hospital of Kunming Medical University, Yunnan Institute of digestive disease, Kunming 650032, China; 2 Department of General Surgery, The First Affiliated Hospital of Kunming Medical University, Yunnan Institute of digestive disease, Kunming 650032, China

## Abstract

The transmembrane receptor guanylate cyclase-C (GC-C) signaling pathway has been implicated in several gastrointestinal disorders. Activation of GC-C via guanylin (Gn) and uroguanylin (Ugn) regulates intestinal fluid and electrolyte homeostasis. However, how it regulates the pathogenesis of inflammatory bowel disease (IBD) is still unclear. Here, we investigated the activation of GC-C signaling in ulcerative colitis (UC) of different clinical severities. A total of 60 UC patients and 20 normal controls were recruited. Evaluation of the UC disease activity index (DAI) was performed using a modified Mayo scoring system. The expression of GC-C, Gn and Ugn in the colonic mucosa was measured by quantitative real-time PCR and Western blot. We found that the UC patients had significantly lower expression of GC-C, Gn and Ugn than the controls. Furthermore, there were significant differences for GC-C, Gn and Ugn expression for the UC groups of Grade 1, 2 and 3, and their expression levels were reduced with increases in their DAI. Taken together, our results demonstrate that GC-C, Gn and Ugn are downregulated in UC, and this downregulation is more significant with aggravation of the clinical condition. Therefore, the GC-C signaling pathway may be implicated in the progression of UC.

Ulcerative colitis (UC) belongs to a group of chronic idiopathic inflammatory disorders that primarily affects the colon and is a major type of inflammatory bowel disease (IBD). Common clinical symptoms in UC are diarrhea, rectal bleeding and abdominal pain. Non-specific manifestations include, among others, fever, loss of appetite and weight loss^1^,^2^. This disease significantly impacts patient quality of life due to its repeated remissions and relapses. There is an increasing incidence rate of IBD in China, particularly of UC^3^,^4^. Although treatment outcome for UC has improved with the use of anti-TNFα agents such as infliximab, the risk of opportunistic infections is increased with such treatments and a certain proportion of patients have severe side effects. Therefore, a continuous search for more specific etiological factors and the identification of novel pharmacological targets are needed.

Recent studies have suggested that the aberrant of guanylate cyclase-C (GC-C) signaling is involved in diarrhea, constipation, abdominal pain, dysfunctional epithelial barrier function, intestinal polyps and tumor growth^5^. GC-C is a transmembrane receptor that is expressed primarily on intestinal epithelial cells (IECs). The peptides guanylin (Gn) and uroguanylin (Ugn) are the endogenous ligands for GC-C, and are highly expressed in the gastrointestinal (GI) epithelium^6^. The binding of these ligands to GC-C results in the conversion of guanosine triphosphate (GTP) to cyclic-guanosine-3′, 5′-monophosphate (cGMP). Increased levels of cGMP activate the cGMP-dependent protein kinase II (PKGII), which phosphorylates the cystic fibrosis transmembrane conductance regulator (CFTR), increasing chloride (Cl^−^) secretion into the lumen. Bicarbonate (HCO_3_^−^) secretion, through an as yet unidentified channel, also occurs in a CFTR-dependent manner. cGMP also inhibits the sodium-hydrogen exchanger NHE3, thereby decreasing sodium (Na^+^) absorption. Therefore, this physiological activation of GC-C regulates intestinal fluid and electrolyte homeostasis, preventing dehydration and intestinal obstruction^7^,^8^,^9^.

A study by Brenna Ø *et al.* revealed that GC-C, Gn and Ugn, as well as several downstream mediators of the GC-C signaling pathway, were all significantly downregulated in both the inflamed colonic mucosa of IBD patients and in rats with 2,4,6-trinitrobenzene sulfonic acid (TNBS) colitis^10^. However, the association between GC-C signaling and the clinical severity of UC has not been previously reported. Therefore, in this study, we investigated the expression of GC-C and its endogenous ligands, Gn and Ugn, in the colonic mucosa of UC patients with different disease activity indexes (DAIs) to evaluate the relationship of the GC-C signaling pathway and UC with different clinical assessments.

## Results

### Clinical parameters in UC patients

As shown in Table 1, there were 18 mildly active UC patients, 23 moderately active UC patients, 19 severely active UC patients, and 20 normal controls in our study. In the UC groups of Grade 1, 2 and 3, the usage rates of 5-aminosalisylic acid (5-ASA) were 73%, 60%, 13%, respectively, and the usage rates of systemic corticosteroids were 18%, 32%, 84%, respectively. There were no significant differences among groups with respect to sex, age and duration of disease.

### mRNA expression of GC-C, Gn and Ugn in mucosal biopsies

**Figure 1:**
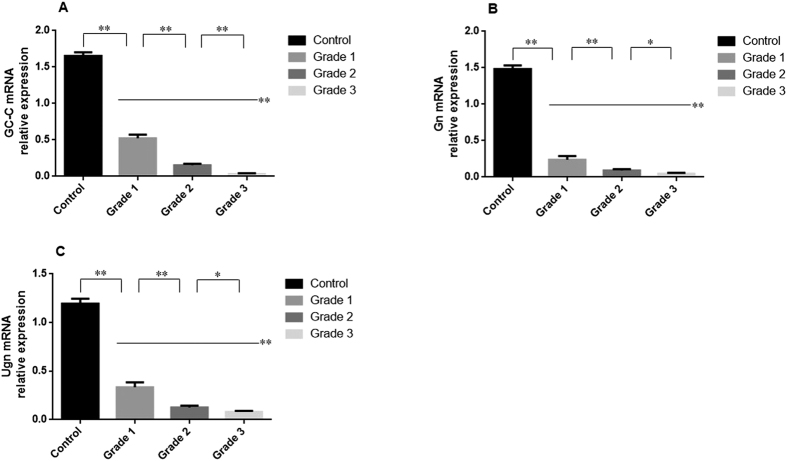
mRNA expression of GC-C, Gn and Ugn in colonic mucosa. The mRNA levels of GC-C (**A**), Gn (**B**) and Ugn (**C**) were determined in the colonic mucosa by qRT-PCR. Control: normal controls (n = 20); Grade 1: mildly active UC group (n = 18); Grade 2: moderately active UC group (n = 23); Grade 3: severely active UC group (n = 19). Gene expression was normalized to the β-actin mRNA levels in each sample. ^*^P < 0.05, ^**^P < 0.01 versus the other groups. Data are expressed as the mean ± SD. The data are representative of three independent experiments from each patient.

As shown in Fig. 1, the mRNA expression levels of GC-C, Gn and Ugn in UC patients were significantly decreased compared with the normal controls (*P* < 0.01). This decrease was more significant with the increase of disease activity. There were significant differences of the GC-C, Gn and Ugn mRNA levels for the UC groups of Grade 1, 2 and 3 (*P* < 0.05).

### Protein levels of GC-C, Gn and Ugn in mucosal biopsies

**Figure 2:**
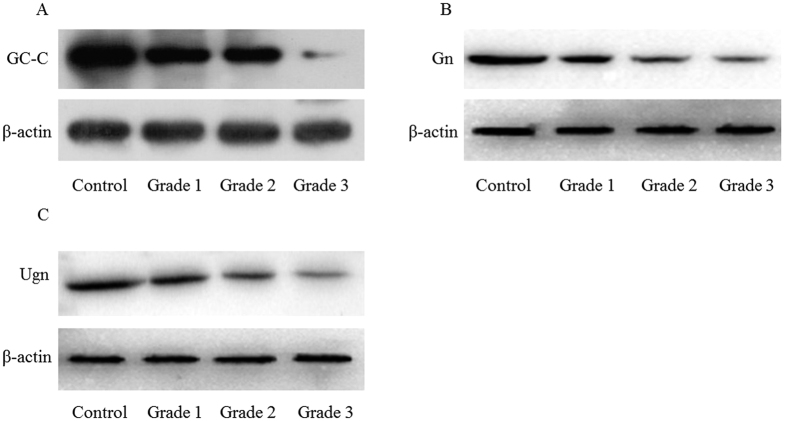
Immunoblot images of GC-C, Gn and Ugn in different groups. The levels of proteins for GC-C (**A**), Gn (**B**) and Ugn (**C**) were detected by Western blot. Groups are shown in Fig. 1. The immunoblot images are representative of each group.

**Figure 3:**
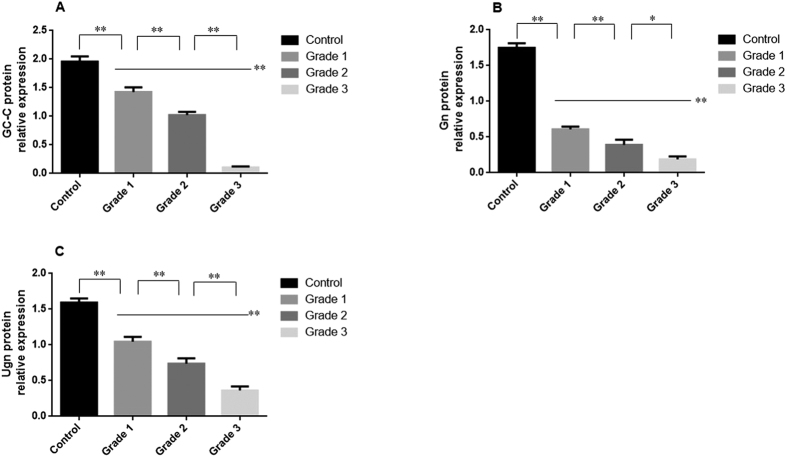
Protein levels of GC-C, Gn and Ugn in the colonic mucosa. The levels of the proteins GC-C (**A**), Gn (**B**) and Ugn (**C**) were determined in the colonic mucosa by Western blot. Groups are shown in Fig. 1. Protein levels were normalized to the β-actin levels in each sample. ^*^P < 0.05, ^**^P < 0.01 versus the other groups. Data are expressed as the mean ± SD. The data are representative of three independent experiments from each patient.

The results of qRT-PCR were confirmed by the protein expression levels, as assessed by western blot analysis. As shown in Figs 2 and 3, the protein levels of GC-C, Gn and Ugn in the UC patients were significantly lower than for the normal controls (*P* < 0.01). This decrease was more significant with the increase of disease activity. There were significant differences for the GC-C, Gn and Ugn protein levels for the UC groups of Grade 1, 2 and 3 (*P* < 0.05).

## Discussion

In the present study, we investigated the expression of GC-C, Gn and Ugn in the inflamed colonic mucosa of UC patients to confirm and extend previous findings. We found that the expression of GC-C and its endogenous ligands, Gn and Ugn, was significantly decreased in UC patients. The combined reduction of both the agonists and their corresponding receptor should lead to a pronounced downstream reduction of GC-C signaling. Similar results were described in a previous report by Brenna Ø *et al.*, showing that the crucial mediators of the GC-C signaling pathway are downregulated in the inflamed colonic mucosa tissues of IBD patients and in rats with TNBS colitis^10^. Furthermore, our findings are the first to demonstrate that the downregulation of GC-C, Gn and Ugn is more significant with an increase of DAI, which suggests that the activation of GC-C signaling is negatively correlated with the clinical severity of UC.

**Figure 4:**
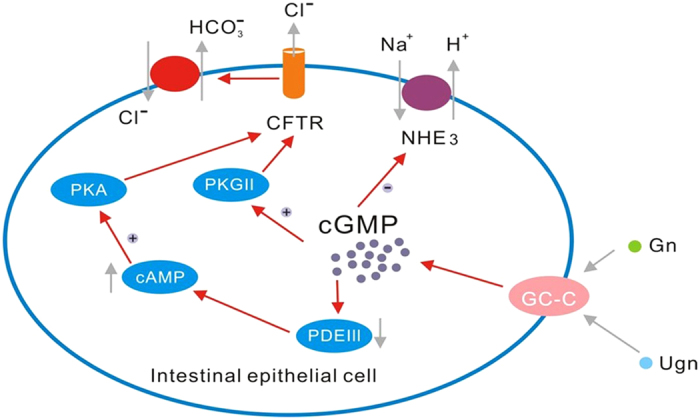
Schematic diagram of the GC-C signaling pathway. Gn and Ugn, endogenous hormones of the gastrointestinal tract, activate GC-C, which is expressed on the surface of intestinal cells along the entire length of the intestines. Gn/Ugn binds to the GC-C receptor resulting in the stimulation of the intracellular production of cGMP. cGMP as a second messenger activates cGMP-dependent PKG II, cross-activates cAMP-dependent protein kinase (PKA) and inhibits a cAMP-specific phosphodiesterase (PDE III). Inhibition of PDE III regulates the action of the apical Na^+^/H^+^ exchanger 3 (NHE3), which causes the decreased absorption of Na^+^. Moreover, PKGII and PKA phosphorylate CFTR, increasing its chloride-secreting activity, exchanging Cl^−^ for HCO_3_^−^, and inducing an increase of bicarbonate secretion. The high concentration of electrolytes in the intestinal lumen results in water influx into the intestine^7^,^8^,^9^.

The GC-C signaling pathway plays a key role in the regulation of intestinal fluid and electrolyte balance^11^. As shown in Fig. 4, endogenous GC-C ligands act as “fluidity sensors” that provide optimal intestinal mucosa hydration through the induction of the net secretion of water, NaCl and HCO_3_^− ^12. A defect in the intestinal barrier function is involved in UC pathogenesis. The mucus layer covering the inner surface of the colorectum provides a complex chemical and physical barrier protecting the host from its harmful external environment, and is composed of approximately 95% water, 5% mucus glycoprotein and small amounts of electrolytes, peptides and lipids^13^. NaCl and HCO_3_^−^ induced by GC-C signaling are the critical electrolytes controlling the rheological properties of the mucus layer and its proper interaction with the adjacent colonic microbiota^14^. Consequently, loss of GC-C signaling may accelerate the progression of intestinal inflammation because of altered transmembrane sodium, chloride and/or bicarbonate movement and the resulting defective water secretion and pH imbalance at the epithelial surface. Additionally, a decrease in the GC-C level causes a reduction in the number of goblet cells that results in the disrupted production of mucus and may cause a loss of mucosal integrity^15^. Moreover, elimination of GC-C or Ugn in mice increased the intestinal permeability through tight junction disassembly, with reduced claudin-2 and JAM-A levels, and the mice were susceptible to chemical-induced colitis^16^,^17^. Collectively, these results suggest that the relationship of the GC-C signaling pathway and intestinal inflammation may be associated with the epithelial barrier function of the intestine.

However, studies on the role of the GC-C signaling pathway in inflammatory gut disorders are controversial. Harml-Laws E *et al.* found that GC-C^−/−^ mice (GC-C knockout mice) had increased proinflammatory gene expression in whole colon tissue and more severe spontaneous colitis when intraperitoneally injected with lipopolysaccharide (LPS)^18^. In a study by Lin *et al.*, GC-C^−/−^ mice had increased susceptibility to colonic inflammatory injury induced by dextran sodium sulfate (DSS)^17^. By contrast, a study by Steinbrecher KA *et al.* showed that DSS-induced clinical disease and histological damage to the colonic mucosa were significantly less severe in GC-C^−/−^ mice and moderately reduced in Gn^−/−^ mice^19^. Fiskerstrand T *et al.* indicated that the increased GC-C signaling disturbed normal bowel function and appeared to have a proinflammatory effect^20^. It seems likely that GC-C signaling regulates intestinal inflammatory responses precisely. GC-C may have differential effects on the development of intestinal inflammation. Thus, the precise mechanism elucidating how the GC-C signaling pathway regulates intestinal inflammatory responses is the basis for ongoing investigation.

In conclusion, our research reveals that the expression of GC-C, Gn and Ugn is reduced in Chinese patients with UC, and the reduction is more significant with the deterioration of the clinical condition. A possible limitation of the current study is the failure to detect the level of cGMP because the experimental materials collected from the patients is limited. The data would also be strengthened if the expression of cGMP in the colonic mucosa of UC patients was assessed. These observations further support the notion that the GC-C signaling pathway may be implicated in the genesis and progression of UC, and the restoration of the dormant GC-C pathway might be a promising treatment for UC.

## Methods

### Ethical considerations

The Institutional Review Board for Clinical Research of the First Affiliated Hospital of Kunming Medical University approved this study, and the methods were performed in accordance with the approved guidelines. Informed consent was also obtained from all subjects.

### Subjects and samples

The UC patients and controls in the study were recruited at the First Affiliated Hospital of Kunming Medical University from 2014 to 2015. The diagnosis of UC was based on clinical, radiological, endoscopic, and histopathological findings in accordance with the Chinese consensus on the diagnosis and management of IBD (2012, Guangzhou, China)^21^. UC disease activity evaluation was performed using a modified Mayo scoring system. The disease activity index (DAI) was assessed by the sum of each variable score in Table 2. Scores ranged from 0 to 12 points. The UC patients are classified into four disease activity subgroups as follows: (1) remission (0≤Mayo score≤2); (2) mildly active disease (3≤Mayo score≤5); (3) moderately active disease (6≤Mayo score≤10); and (4) severely active disease (11≤Mayo score≤12)^22^. The subjects included 18 mildly active UC patients (Grade 1), 23 moderately active UC patients (Grade 2) and 19 severely active UC patients (Grade 3). Patients without macroscopic or histopathological abnormalities and with no evidence for underlying GI pathology served as controls. All participants underwent colonoscopy, and all endoscopic pinch biopsies were collected from the mucosa of the sigmoid colon. Biopsies were taken at the sites of active inflammation adjacent to the ulcerations or normal mucosa according to the experimental requirements. Six biopsy specimens were taken from each patient. Of these, three biopsy specimens per patient were used for the qRT-PCR study, and the other three specimens were used for Western blot analysis. Endoscopic biopsies used for qRT-PCR were immediately placed in RNAlater (Qiagen, Hilden, Germany) and stored at −80 °C until processing. Biopsy samples used for Western blotting were immediately placed into liquid nitrogen and then transferred to −80 °C freezer to ascertain the quality of the material.

### Quantitative real-time PCR analysis

Quantitative real-time polymerase chain reaction (qRT-PCR) was performed to assess the mRNA expression of Gn, Ugn and GC-C. One target gene was examined from each biopsy specimen per patient, and the experiment was performed three times. Total RNA was extracted from the freshly frozen biopsies using Trizol reagent (Qiagen, Hilden, Germany). The concentration and purity of the obtained RNA were verified spectrophotometrically at 260/280 nm, and the integrity was validated by agarose gel electrophoresis. The complementary DNA was synthesized using SYBR PrimeScript RT reagent kits (TaKaRa, Dalian, China) according to the manufacturer’s instructions. Quantitative real-time PCR was performed in an ABI prism 7900 HT sequence detector (Applied Biosystems, Foster City, CA, USA) using the SYBR green methodology. β-actin was used as the endogenous reference gene. Briefly, in a 20 μl reaction volume, 1 μl of cDNA was added to 10 μl of SYBR green Master mix (Darmstadt, Germany) and 0.3 μmol/L of each primer. The specific primers of GC-C (#HQP008552) and Gn (#HQP008543) were purchased from GeneCopoeia (Guangzhou, China). The specific primers for Ugn and β-actin are as follows: Ugn, F, 5′- GTACCCTGAGCCCACCAG-3′ and R, 5′-CGATTGCTTTGTACCAGATCC-3′; β-actin, F, 5′- CCAGGGCGTTATGGTA GGCA-3′ and R, 5′-TTCCATATCGTCCCAGTTGGT-3′ (Jierui, Shanghai, China). The conditions for the PCR reactions were identical and were as follows: 95 °C 10 min; 95 °C 15 sec, 60 °C 30 sec, 40 cycles; 65 °C 5 sec; and 95 °C. The lengths of expected products were as follows: GC-C (146 bp); Gn (93 bp); Ugn (150 bp); β-actin (130 bp). The comparative 2^−ΔΔCT^ method was used to calculate the relative expression level of each target gene with β-actin as the internal control. Quantitative real-time PCR analysis was performed as described previously^23^,^24^.

### Western blot analysis

Western blot was performed to assess the protein levels of Gn, Ugn, and GC-C. One target gene was examined from each biopsy specimen per patient, and the experiment was performed three times. The total protein from the mucosal samples was extracted using radioimmunoprecipitation assay (RIPA) protein lysis buffer that contained protease inhibitor (Beyotime Institute of Biotechnology, Shanghai, China). Protein was quantified using a BCA protein quantification kit (Beyotime). Briefly, equal amounts of protein were loaded, and electrophoresis was applied on a 12% sodium dodecyl sulfate-polyacrylamide gel electrophoresis (SDS-PAGE). Proteins were transferred to polyvinylidene difluoride (PVDF) membranes and blocked with 5% fat-free milk at room temperature for 1 h. Then, the membranes were incubated with primary antibody (1:1000 for mouse monoclonal anti-GC-C, Sigma; 1:250 for rabbit polyclonal anti-Gn, Abcam; 1:500 for goat polyclonal anti-Ugn, Santa; and 1:5000 for anti-β-actin, Millipore) overnight at 4 °C. After three washes in TBST (10 min each), the membranes were incubated with horseradish peroxidase (HRP)-conjugated secondary antibody (1:5000 for goat anti-mouse IgG, Lianke, China) for 1 h at room temperature. After three washes in TBST (10 min each), the protein bands were visualized using an enhanced chemiluminescence reagent (ECL kit, Amersham, UK) and X-ray film (Kodak, USA). Densitometry of the protein bands was analyzed with the Image J software. β-actin was used as the internal reference.

### Statistics

All data were expressed as the mean ± standard deviation (SD), and normality tests were performed using the Kolmogorov-Smirnov test. Variables with a non-normal distribution were logarithmically transformed before statistical analysis. Data were analyzed using one-way analysis of variance (ANOVA) followed by LSD-t post hoc tests or the independent sample t-test, as appropriate. A P < 0.05 was regarded as statistically significant. All statistical analyses were performed using the SPSS 17.0 software (Chicago, USA).

## Additional Information

**How to cite this article**: Lan, D. *et al.* Expression of guanylate cyclase-C, guanylin, and uroguanylin is downregulated proportionally to the ulcerative colitis disease activity index. *Sci. Rep.* **6**, 25034; doi: 10.1038/srep25034 (2016).

## Figures and Tables

**Table 1:** Number and clinical characteristics of the subjects.

	Control	Grade 1	Grade 2	Grade 3
Subjects	20	18	23	19
Sex(male/female)	9/11	10/8	11/12	10/9
Age (years)	48.15 ± 8.95	46.50 ± 13.49	44.30 ± 10.03	44.11 ± 10.33
Duration of disease (years)		2.78 ± 1.59	2.65 ± 1.37	3.58 ± 1.77
5-ASA usage (%)		73%	60%	13%
Corticosteroid usage (%)		18%	32%	84%

**Table 2:** Modified Mayo Disease Activity Index.

Grade	Stool frequency	Rectal bleeding	Physician’s global assessment	Endoscopy finding
0	Normal number of stools per day for this patient	No blood seen	Normal	Normal or inactive disease
1	1 or 2 more stools than normal	Streaks of blood with stool less than half the time	Mild disease	Mild disease (erythema, decreased vascular pattern)
2	3 or 4 more stools than normal	Obvious blood with stool most of the time	Moderate disease	Moderate disease (marked erythema, absent vascular pattern, friability, erosions)
3	5 or more stools than normal	Blood alone passed	Severe disease	Severe disease (spontaneous bleeding, ulceration)
